# Association Between Dietary Folate and Prostate Cancer Aggressiveness Among African Americans and European Americans

**DOI:** 10.3390/nu18050748

**Published:** 2026-02-26

**Authors:** Lihchyun Joseph Su, Sarah O’Connor, Daniela Ramirez Aguilar, MinJae Lee, Harrison Wong, Hui-Yi Lin, Jeannette T. Bensen, James L. Mohler, Lenore Arab, Longgang Zhao, Ebonee N. Butler, Laura Farnan, Susan E. Steck

**Affiliations:** 1 Peter O’Donnell Jr. School of Public Health, University of Texas Southwestern Medical Center, Dallas, TX 75390, USAminjae.lee@uth.tmc.edu (M.L.); 2 Fay W. Boozman College of Public Health, University of Arkansas for Medical Sciences, Little Rock, AR 72205, USA; 3 School of Public Health, Louisiana State University Health Sciences Center, New Orleans, LA 70112, USA; hlin1@lsuhsc.edu; 4 Gillings School of Global Public Health, University of North Carolina, Chapel Hill, NC 27599, USA; jeannette_bensen@med.unc.edu (J.T.B.); ebonee@live.unc.edu (E.N.B.); laura_farnan@med.unc.edu (L.F.); 5 Department of Urology and Lineberger Comprehensive Cancer Center, University of North Carolina, Chapel Hill, NC 27599, USA; jamesmohler612@gmail.com; 6 David Geffen School of Medicine, University of California, Los Angeles, CA 90024, USA; larab@mednet.ucla.edu; 7 Arnold School of Public Health, University of South Carolina, Columbia, SC 29208, USA; longgang.zhao@yale.edu (L.Z.); stecks@mailbox.sc.edu (S.E.S.)

**Keywords:** prostate cancer, tumor aggressiveness, folate, racial disparity

## Abstract

**Background:** Despite the confirmed beneficial effects on preventing neural tube defects, concerns about high intakes of synthetic folate, or folic acid, in promoting cancer progression have been raised. This study evaluated the association between folate intake and prostate cancer (PCa) aggressiveness among African-American (AA) and European-American (EA) males. **Methods:** This study included 722 AA and 775 EA men with prostate cancer. Folate intake (dietary folate equivalent (DFE), synthetic folate, natural folate) was estimated using the National Cancer Institute Dietary History Questionnaire and detailed dietary supplement use questionnaire. Analyses included univariable comparisons of demographic and clinical characteristics of the two racial groups using the *t*-test or its non-parametric counterpart, the Wilcoxon test for continuous variables, and the Chi-square test for categorical variables. Logistic regression analysis was performed to evaluate the associations of each source of folate intake with PCa aggressiveness. Interaction effects between folate intake levels and racial groups were tested to evaluate if the association between folate intake and PCa differed by racial groups. **Results:** A greater proportion of AA subjects were diagnosed with high PCa aggressiveness compared to EAs (31.6% vs. 21.7%; *p* < 0.001). Both AAs and EAs had associations between decreased DFE intake and PCa aggressiveness after adjusting for covariates. Among AAs, men with the highest quartile levels of synthetic folate intake had higher odds of high-aggressive PCa compared to those with the lowest levels of intake (adj. OR = 1.39; *p* = 0.27), while the reversed association became stronger among EAs (adj. OR = 0.62; *p* = 0.14). **Conclusions:** The association between folate intake and prostate cancer aggressiveness appears to be source-specific and modified by race. These findings highlight the need for population-informed nutritional guidance and further investigation into nutrient–gene and dietary pattern interactions in prostate cancer progression.

## 1. Introduction

Prostate cancer (PCa) is among the leading cancers in incidence and mortality among males in the United States (US). The majority of PCa cases are of the indolent type, thus not requiring treatment. However, about 30% of PCa cases are aggressive, with a high risk of progressing to lethal metastatic disease [1,2]. African-American (AA) men are more likely to be diagnosed with the aggressive form of the disease when compared to other racial groups. Previous studies have identified racial disparities in PCa cases between AA and European-American (EA) men [3,4,5,6,7,8,9]. AA have an incidence rate 1.5 times higher and a mortality rate more than double that of EA [2,10]. Additionally, AA are diagnosed with a more aggressive form of the disease at a younger age compared to other racial groups, which cannot be explained entirely by differences in either socioeconomic status or access to care [11].

In 1998, the US Food and Drug Administration required products, such as orange juice, bread, flour, cornmeal, rice, pasta, and other grain products, to be enriched with folic acid to prevent fetal neural tube defects [12]. The recommended daily intake is 400 mcg of synthetic folate for females of reproductive age, with an upper limit for natural folate of 1000 mcg per day [10,13]. Folate is a water-soluble B vitamin found naturally in foods like dark-green leafy vegetables and legumes. Low folate availability has been studied and shown to influence cancer development through altered methylation patterns, as folate is a critical component of one-carbon metabolism required for the synthesis of S-adenosylmethionine, the primary methyl donor for DNA methylation [14]. Inadequate folate status may lead to global DNA hypomethylation, aberrant gene expression, and genomic instability, thereby promoting carcinogenesis. Its synthetic form, folic acid, is present in supplements and fortified foods, such as grains and cereals [15]. Dietary folate is the reduced state of polyglutamate acids side chains requiring oxidation and hydrolysis for absorption. In contrast, folic acid is the oxidized form of pteroylmonoglutamate, which makes it readily available. Dietary folate bioavailability can range from 10 to 98% and is influenced by intestinal pH, enzymatic activity, the presence of alcohol and other inhibitors, including certain medications (e.g., methotrexate and anticonvulsants) [16], gastrointestinal conditions that impair absorption (e.g., celiac disease and inflammatory bowel disease) [17], and interactions with other dietary components, malabsorption disorders, and the food matrix.) [18]. Folate equivalents, or dietary folate equivalents (DFEs), account for the differences in absorption between folate and folic acid. For example, one mcg of DFE equals one mcg of dietary folate, 0.6 mcg of folic acid consumed with food, or 0.5 mcg of supplemental folic acid on an empty stomach [10]. Many countries, including the US, fortify food products with the synthetic form of folate or folic acid. Notably, epidemiologic evidence to date has been largely null. A meta-analysis published in Prostate Cancer and Prostatic Diseases in 2014 reported that neither dietary folate nor total folate intake was significantly associated with overall prostate cancer risk, underscoring the inconsistency of findings linking folate exposure to prostate carcinogenesis [19]. This study utilized a population-based case-only design. It evaluated the association between dietary folate intake in the year preceding PCa diagnosis and PCa aggressiveness among AA and EA.

## 2. Materials and Methods

### 2.1. Study Participants

The North Carolina-Louisiana Prostate Cancer Project (PCaP) is a population-based, cross-sectional, case-only, incident PCa study investigating racial and geographical differences in PCa aggressiveness [7]. A rapid case ascertainment system was used to identify men with a first diagnosis of histologically confirmed adenocarcinoma of the prostate in North Carolina (NC) and Louisiana (LA) between 1 July 2004, and 31 August 2009. Residents of NC and LA were eligible if they resided within the study catchment areas and were (1) 40–79 years old at diagnosis; (2) self-identified as AA/Black or “Caucasian”/EA/White; (3) able to complete the study interview in English; (4) not living in an institution (e.g., nursing home); (5) mentally and physically able to complete the interview. Written informed consent was obtained from each study participant before participation. Approximately equal numbers of AAs and EAs were enrolled from NC (AA *n* = 505; EA *n* = 527) and LA (AA *n* = 632; EA *n* = 603), with participation rates of 62% in NC, 72% in pre-Hurricane Katrina LA, and 63% in post-Hurricane Katrina LA. Further details of the methods and designs of PCaP have been published [7]. The analytic population comprised 2173 PCaP study participants with complete data on PCa tumor aggressiveness. Before data analysis, study participants with intermediate PCa tumor aggressiveness were excluded to avoid misclassification for PCa aggressiveness, leaving a final study sample of 1497 (AA *n* = 722, EA *n* = 775). Written informed consent was obtained from all participants prior to data collection, including consent for analysis and publication of de-identified data. The study protocol and data-processing procedures were approved by the Institutional Review Boards of the University of North Carolina at Chapel Hill, Louisiana State University Health Sciences Center, and the Department of Defense Prostate Cancer Research Program (approval code: NHD 03262023, approval date: 26 March 2023). Additional IRB reviews were obtained at the University of Texas Southwestern Medical Center and the University of Arkansas for Medical Sciences for the secondary data analysis for this manuscript.

### 2.2. Study Variables and Definition of Tumor Aggressiveness

Consenting study participants completed structured, in-home, interviewer-administered questionnaires that included information on demographics, pre-diagnostic PCa screening history, comorbidities, family health history, healthcare access, and behavioral factors, such as physical activity, alcoholic beverage consumption, and smoking status (https://pcap.bioinf.unc.edu/interviews.php (accessed on 22 July 2025)). The interviewers, who were research nurses trained explicitly for data collection, also obtained anthropometric measurements (e.g., height, weight) using a standardized protocol at the end of each interview. Data on clinical attributes of PCa, including cancer stage at diagnosis, Gleason sum, and prostate-specific antigen (PSA) level at diagnosis, were abstracted from study participants’ medical records obtained from diagnosing physicians. The medical records abstraction was performed by trained personnel, and a random sample of the abstracted medical records (approximately 10%) was abstracted a second time by another staff member to ensure abstractor consistency. In PCaP, PCa aggressiveness was defined by a combination of Gleason sum, clinical stage, and PSA level at diagnosis as follows: (1) high aggressive (Gleason sum ≥ 8 or PSA > 20 ng/mL, or Gleason sum ≥ 7 and clinical stage T3–T4); (2) low aggressive (Gleason sum < 7 and clinical stage T1–T2 and PSA <10 ng/mL); (3) intermediate aggressive PCa (all others). A case–case study design was adopted for the present analyses to contrast study participants with high-aggressive PCa to those with low-aggressive PCa. Participants with intermediate PCa were excluded from the analysis to avoid potential misclassification.

### 2.3. Dietary Assessment and Supplement Use

Dietary data were obtained using the National Cancer Institute (NCI) Dietary History Food Frequency Questionnaire [20] that was modified to include the regional dishes of NC and LA. The modified 144-item questionnaire included questions on the frequency of food intake, usual portion size, and food preparation methods in the 12 months before diagnosis with PCa. Information on dietary supplement use was solicited via a validated questionnaire [21] administered by the research nurses during in-home visits. Data on supplemental folic acid intake were derived from responses to questions about using multivitamins containing folic acid and single-nutrient folic acid supplements. For multivitamins, study participants were asked whether they had taken multivitamin supplements in the 12 months before PCa diagnosis (no, less than once a week, or yes) and, if yes, the frequency of use (1–2, 3–4, 5–6, 7 days/week). Forty-five percent of the study participants reported multivitamin supplement use in the previous 12 months. They were asked to identify the most often used brand from a list of common multivitamin brands in the US, which included an open-ended option for unlisted brands. Subsequently, these study participants were asked to provide the study nurse with a multivitamin supplement bottle for recording nutrient contents and doses. Study participants who could not provide the multivitamin bottle (about 5% of users) were assigned the folic acid dose listed on the manufacturer’s label of the stated brand. When the manufacturer’s label (less than 1%) could not be found, study participants were assigned the folic acid dose of the most used brand among multivitamin supplement users (i.e., Centrum); this value was 100 mcg. In subsequent questions, study participants were asked about using single-nutrient supplements and, if yes (13% of subjects), the frequency of use (same categories as above). Study participants who could not provide the supplement bottle were asked to indicate the usual dose. Dose choices for single-nutrient folic acid supplements were 100, 200, 400, 800, or 1000 mcg/day, and an open-ended option for unlisted doses. Study participants who reported using single-nutrient folic acid supplements but could not provide the supplement bottle or were unable to report the usual dose (4% of users) were assigned the mode dose (i.e., 400 mcg) among single-nutrient folic acid supplement users.

### 2.4. Statistical Analysis

Univariable comparisons were conducted on demographic and clinical characteristics of the two racial groups using the *t*-test or its non-parametric counterpart, the Wilcoxon test for continuous variables, and the Chi-square test for categorical variables. The associations of each source of folate intake with a binary outcome variable (i.e., prostate cancer aggressiveness [high vs. low]) were evaluated using multivariable logistic regression analysis. Specifically, interaction effects between folate intake levels and racial groups were tested to determine whether the association between folate intake and prostate cancer aggressiveness differed by racial group. Three forms of folate were identified: natural, synthetic, and dietary folate equivalent (DFE). All folate exposure variables were categorized into quartiles based on the distribution of the high-aggressive group. Because the distributions of dietary folate intake were quite different between these two groups. There were too few subjects in some cells if “low-aggressive group” is used to define the quartile. In selecting the multivariable-adjusted models, the following variables were considered to be potential confounders based on a review of the literature: age (continuous); previous screening (no PSA test, only pre-diagnostic digital rectal exam, only pre-diagnosis PSA test, or pre-diagnosis PSA test and digital rectal exam); first-degree relative with PCa (yes/no); smoking status (never, former, current); education (less than high school graduate, high school graduate, more than high school graduate, more than college graduate); body mass index (BMI: kg/m^2^, continuous); and total energy intake (kcal/day). Additionally, this analysis applied the residual method for saturated fat (continuous) and total energy, where saturated fat was dependent on the total energy intake [22]. For each model, we evaluated underlying assumptions, including linearity. All statistical analyses were performed using SAS^®^ version 9.4 (SAS Institute, Inc., Cary, NC, USA) with statistical significance set at α = 0.05 (two-tailed) [23].

## 3. Results

### 3.1. Baseline Characteristics

Among 1497 PCa study participants included in the analyses, 722, or 48% were AA. Various characteristics between AA and EA were compared in Table 1. AA were younger (62 vs. 64 years old; *p* < 0.001), reported higher intakes of energy (2847 vs. 2370 Kcal; *p* < 0.001) and residual saturated fat intake (0.22 vs. −0.2; *p* < 0.001), included a more significant proportion of current smokers (22.1% vs. 9.3%) and an education level less than high school graduates (33.6% vs. 9.7%), and more often reported having undergone PSA screening (20.8% vs. 6.3%) and pre-diagnosis digital rectal exam. A higher proportion of AA were diagnosed with high-aggressive PCa compared to EAs (31.6% vs. 21.7%; *p* < 0.001).

### 3.2. Interactive Multivariable Analysis

**Figure 1:**
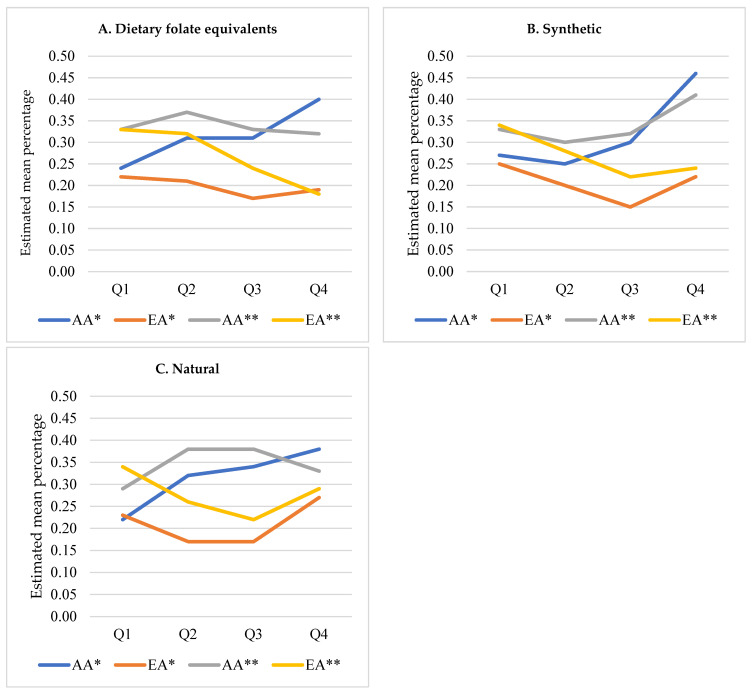
Adjusted mean percentage of high-aggressive prostate cancer across quartiles of dietary folate intake by race. AA = African American; EA = European American. Estimates were derived from multivariable logistic regression models adjusted for age, body mass index, prior screening, smoking status, education, family history of prostate cancer, total energy intake, and residual saturated fat intake (* Adjusted for age and body mass index (BMI); ** Adjusted for age, BMI, previous screening, smoking status, education, first-degree relative diagnosed with PCa, total energy, and the residual (continuous) saturated fat intake).

As shown in Table 2, associations of each source of folate intake (DFE, synthetic, and natural) with high-aggressive PCa were observed and differed by racial groups in multivariable models after adjusting for age and BMI (overall *p*-value of interaction effect between quartile levels and race was *p* = 0.05 for DFE, *p* = 0.02 for both synthetic and natural). AA study participants with the highest quartile levels of intake had higher odds of high-aggressive PCa compared to those with the lowest levels of intake (adjusted odds ratio (adj. OR) = 2.09; *p* = 0.002 for DFE, 2.25; *p* = 0.001 for synthetic, 2.19; *p* = 0.001 for natural), while these findings were either reversed (adj. OR = 0.82; *p* = 0.47 for DFE, 0.84; *p* = 0.54 for synthetic) or attenuated (adj. OR = 1.22; *p* = 0.46 for natural) among EA. The adjusted mean percentages of high-aggressive PCa over quartile levels of each intake by racial group were calculated based on multivariable models and were plotted in Figure 1.

As shown in Figure 1, the directionality for the point estimates was quite different, whether adjusting for age and body mass index versus more adjusted models. Therefore, fully adjusted models were used in Table 3. Among AA, study participants with the highest quartile levels of DFE intake no longer had higher odds of high-aggressive PCa compared to those with the lowest levels of intake (adjusted odds ratio (adj. OR = 0.96; *p* = 0.9), and the findings among EAs became stronger (adj. OR = 0.45; *p* = 0.02) compared to those from the model with an adjustment of only age and BMI. As shown in Figure 1, both AA and EA had associations of decreased DFE intake with PCa aggressiveness after further adjustment. For natural folate intake, the association of increased intake with PCa aggressiveness was no longer found among AA after adjusting for additional variables (Figure 1C). The findings of synthetic folate intake remained similar (Figure 1B); among AA, study participants with the highest quartile levels of intake had higher odds of high-aggressive PCa compared to those with the lowest levels of intake (adj. OR = 1.39; *p* = 0.27), while reversed association became stronger among EA (adj. OR = 0.62; *p* = 0.14). The interaction effects we found in the models with an adjustment of age and BMI were attenuated (*p* = 0.25 for DFE, *p* = 0.14 for synthetic, and *p* = 0.03 for natural folate intake).

## 4. Discussion

The findings of this study suggest a complex interaction between dietary folate intake and prostate cancer aggressiveness, which appears to be modulated by race and dietary patterns, particularly among AA men. As depicted in Figure 1, there is a pattern where increased dietary folate, primarily from natural sources, such as collard greens, a staple in AA diets in the South, does not correlate with decreased PCa aggressiveness after adjusting for total caloric intake and saturated fat (Table 3). This finding is critical because collard greens are typically cooked and soaked in animal fats, which are high in saturated fats known to be associated with PCa. Thus, while the crude model initially suggested a protective effect of high folate intake, this association was attenuated after these adjustments, which suggests potential confounding by saturated fat intake. Moreover, given the strong correlation between saturated fat and PCa, it is plausible that residual confounding could still be influencing these associations, which underscores the complexity of dietary impacts on health outcomes in racially diverse populations.

Established risk factors for PCa include advanced age, race and ethnicity, and family history. Modifiable risk factors positively associated with PCa include inflammation, hyperglycemia, infections, environmental exposure to chemicals or ionizing radiation, obesity, physical inactivity, and certain dietary factors [24]. The findings observed in the present study are consistent with those included in a 2012 meta-analysis that identified ten randomized clinical trials that showed a significant increase in overall cancer frequency among the group who took folic acid compared to the controls [11]. While several previously conducted studies also supported these findings, evidence of no association or a minor increase in risk has also been reported. However, it is essential to note that studies with little or no results of an association included mostly EA men and cannot be generalized to the AA population, who have a more significant burden of aggressive PCa than other racial and ethnic groups [14]. These results further demonstrate the disparate impact that folate intake may have according to race and highlight the need for future research that includes diverse study populations.

Genetic variation between populations of African and European ancestry may also contribute to the differential associations observed in this study. Prior literature has documented population-specific differences in the frequency of polymorphisms in folate-metabolizing genes, including MTHFR [25,26,27], MTR [28,29], MTRR [29,30], and DHFR [31,32], which influence folate bioavailability [33], one-carbon metabolism [34,35], and DNA methylation [31,36,37]. In addition, prostate cancer susceptibility loci, including HOXB13 [38,39,40] and regions on chromosome 8q24 [41,42], have been shown to vary in allele frequency between AA and EA populations and have been associated with PCa risk and aggressiveness. Although genetic data were not available in the present analysis, these nutrient–gene interactions represent a biologically plausible mechanism through which folate intake may differentially influence PCa aggressiveness across racial groups and warrant further investigation.

The differential impact of folate intake on PCa aggressiveness, particularly between AA and EA populations, aligns with and expands upon previously reported racial disparities in disease severity and outcomes [32,43,44,45]. This study contributes to the ongoing discourse by illustrating that the association between folate intake and prostate cancer aggressiveness may be confounded by dietary habits specific to certain racial groups, such as the preparation methods of collard greens in AA diets. These insights underscore the necessity of modifying public health guidelines that consider racial and ethnic dietary patterns when addressing nutrient intake and cancer risk.

Furthermore, based on epidemiological and experimental evidence, concern has arisen that high synthetic folate intake, which can occur upon consuming many multivitamins and supplements, may promote cancer progression [14,24,46,47,48,49]. For example, folic acid is commonly consumed in combination with vitamins B6 and B12 in multivitamin formulations [50], which together influence one-carbon metabolism and DNA methylation. Prior studies have reported that high-dose folic acid supplementation, particularly in combination with B vitamins, may promote the progression of pre-existing neoplastic lesions [51,52,53,54,55]. The proposed pathway for excessive folic acid carcinogenesis is an increase in unmetabolized synthetic folate on natural killer cells that leads to a decrease in the destruction of tumor cells [15]. These findings lend further support to concerns that high synthetic folate intake may increase the odds of prostate cancer aggressiveness, particularly among AA men.

Overall, this study has several strengths. The sample population was optimized for studying population-based racial differences in PCa aggressiveness because recruitment was based on race, and similar numbers of AA and EA were enrolled. Enrolling only men with histologically confirmed PCa and using low-aggressive cases as the comparison group may have minimized outcome misclassification due to the high prevalence of indolent PCa in the American population. In traditional case–control studies, some “controls” may be erroneously classified as disease-free because they had not been previously screened. Data on potential confounders and effect modifiers were collected from study participants and used in the analyses. However, no observational study can rule out residual or unmeasured confounding.

This research is subject to several limitations. The NCI Dietary History Questionnaire, modified to include regional dishes of NC and LA, assessed the study participants’ diet for the year before diagnosis. Food frequency questionnaires can be inaccurate and are innately subject to recall bias; however, the effect of differential recall bias is likely minimized in this study because all study participants were diagnosed with PCa [22]. Even assuming the accuracy of dietary information, only food intakes for the year before diagnosis were assessed. However, the most relevant period of dietary exposure for PCa is unknown, but diets are likely to be relatively stable in adulthood [56,57]. Fatigue is a known issue with completing long dietary history questionnaires, which can negatively impact the accuracy and completeness of the data collected. PCaP questionnaires were completed by trained personnel via interview. Breaks and prompts were provided to study participants to reduce the impact.

## 5. Conclusions

The results of this study suggest an inverse association between consuming a low-to-moderate quantity of folate, i.e., around or below the recommended daily amount, against highly aggressive PCa. Associations between increased folate intakes and low PCa aggressiveness were observed among EA subjects. Among AAs, the association between dietary folate and highly aggressive PCa is not evident, although there is a general positive direction. Future research is needed to corroborate these findings and to understand better how additional factors may influence racial disparities in PCa aggressiveness.

## Figures and Tables

**Table 1:** Distribution of demographic and clinical characteristics by race and PCa aggressiveness among PCaP study participants.

Characteristics	All (*n* = 1497)	African American (*n* = 722)	European American (*n* = 775)	*p*-Value ^1^
	Mean (SD)	Mean (SD)	Mean (SD)	
Age at diagnosis, y	63 (8)	62 (8)	64 (8)	<0.001
Body mass index, kg/m^2^	29.20 (5.44)	29.16 (5.92)	29.25 (4.95)	0.748
Total food energy, kcal, mean(SD)	2600 (1420)	2847 (1638)	2370 (1135)	<0.001
Synthetic folic acid intake, mcg/d, median (IQR)	145.2 (96.20, 213.5)	149.0 (96.87, 240.1)	139.0 (95.76, 198.1)	0.029
Dietary folate equivalents intake (DFE), mcg/d, median (IQR)	544.9 (403.2, 750.4)	574.2 (405.2, 825.7)	527.0 (402.5, 694.8)	<0.001
Natural folate intake, mcg/d, median (IQR)	289.4 (206.4, 407.7)	306.2 (213.9, 443.9)	276.1 (201.8, 370.2)	<0.001
PCa aggressiveness, high, *n* (%)	396 (26.45)	228 (31.58)	168 (21.68)	<0.001
First-degree relative diagnosed with PCa, yes, *n* (%)	338 (24.49)	167 (25.38)	171 (23.68)	0.464
**Education level, *n* (%)**				<0.001
Less than a high school graduate	317 (21.20)	242 (33.61)	75 (9.68)	
High school graduate	360 (24.08)	191 (26.53)	169 (21.81)	
More than high school graduate	385 (25.75)	182 (25.28)	203 (26.19)	
More than college graduate	433 (28.96)	105 (14.58)	328 (42.32)	
**Smoking status, *n* (%)**				<0.001
Never smoker	504 (33.69)	212 (29.40)	292 (37.68)	
Former smoker	761 (50.87)	350 (48.54)	411 (53.03)	
Current smoker	231 (15.44)	159 (22.05)	72 (9.29)	
**Previous screening, *n* (%)**				<0.001
No PSA test or pre-diagnosis digital rectal exam	199 (13.29)	150 (20.78)	49 (6.32)	
Only pre-diagnosis digital rectal exam	244 (16.30)	155 (21.47)	89 (11.48)	
Only pre-diagnosis PSA test	64 (4.28)	29 (4.02)	35 (4.52)	
Pre-diagnosis PSA test and digitalrectal exam	990 (66.13)	388 (53.74)	602 (77.68)	
**Synthetic folic acid intake, quartile ^2^**				<0.001
Q1 (<92.6 mcg/d)	347 (23.18)	166 (22.09)	181 (23.35)	
Q2 (92.6–150.2 mcg/d)	436 (29.12)	196 (27.15)	240 (30.97)	
Q3 (150.2–243.1 mcg/d)	432 (28.86)	186 (25.76)	246 (31.74)	
Q4 (>243.1 mcg/d)	282 (18.84)	174 (24.10)	108 (13.94)	
**Dietary folate equivalents intake (DFE), quartile ^2^**				<0.001
Q1 (<409.1 mcg/d)	390 (26.05)	182 (25.62)	205 (26.45)	
Q2 (409.1–553.0 mcg/d)	375 (25.05)	160 (22.16)	215 (27.74)	
Q3 (553.0–810.3 mcg/d)	416 (27.79)	185 (25.62)	231 (29.81)	
Q4 (>810.3 mcg/d)	316 (21.11)	192 (26.59)	124 (16.00)	
**Natural folate intake, quartile ^2^**				<0.001
Q1 (<209.2 mcg/d)	392 (26.19)	174 (24.10)	218 (28.13)	
Q2 (209.2–303.1 mcg/d)	408 (27.25)	180 (24.93)	228 (29.42)	
Q3 (303.1–435.6 mcg/d)	390 (26.05)	180 (24.93)	210 (27.10)	
Q4 (>435.6 mcg/d)	307 (20.51)	188 (26.04)	119 (15.35)	

^1^ Chi-square test for categorical variables; *t*-test or Wilcoxon test for continuous variables. ^2^ Based on the high-aggressive PCa group.

**Table 2:** Crude model adjusting for age and body mass index, evaluating ingestion of dietary folate equivalents, synthetic and natural folate levels by race ^4^, and level of PCa aggressiveness.

	African Americans				European Americans			
	OR ^1^/95% CI ^2^	*p*-Value	Adjusted Mean	SE ^3^ of Adjusted Mean	OR/95% CI	*p*-Value	Adjusted Mean	SE of Adjusted Mean
**Dietary folate equivalents (DFEs)**								
Q1 (<409.1 mcg/d)	1.00		0.24	0.032	1.00		0.22	0.029
Q2 (409.1–553.0 mcg/d)	1.37 (0.84, 2.23)	0.213	0.31	0.038	0.94 (0.60, 1.48)	0.791	0.21	0.028
Q3 (553.0–810.3 mcg/d)	1.36 (0.85, 2.18)	0.194	0.31	0.035	0.71 (0.45, 1.14)	0.162	0.17	0.025
Q4 (>810.3 mcg/d)	2.09 (1.33, 3.30)	0.002	0.40	0.037	0.82 (0.47, 1.41)	0.468	0.19	0.035
**Synthetic**								
Q1 (<92.6 mcg/d)	1.00		0.27	0.035	1.00		0.25	0.032
Q2 (92.6–150.2 mcg/d)	0.89 (0.55, 1.45)	0.645	0.25	0.032	0.74 (0.47, 1.16)	0.188	0.20	0.025
Q3 (150.2–243.1 mcg/d)	1.15 (0.72, 1.85)	0.555	0.30	0.034	0.54 (0.34, 0.87)	0.012	0.15	0.023
Q4 (>243.1 mcg/d)	2.25 (1.41, 3.60)	0.001	0.46	0.039	0.84 (0.48, 1.47)	0.544	0.22	0.040
**Natural**								
Q1 (<209.2 mcg/d)	1.00		0.22	0.032	1.00		0.23	0.029
Q2 (209.2–303.1 mcg/d)	1.62 (0.99, 2.64)	0.054	0.32	0.036	0.66 (0.42, 1.05)	0.077	0.17	0.024
Q3 (303.1–435.6 mcg/d)	1.80 (1.11, 2.92)	0.018	0.34	0.036	0.66 (0.41, 1.05)	0.082	0.17	0.025
Q4 (>435.6 mcg/d)	2.19 (1.35, 3.53)	0.001	0.38	0.037	1.22 (0.72, 2.05)	0.462	0.27	0.042

^1^ Odds ratio. ^2^ Confidence interval. ^3^ Standard error. ^4^ Interaction effect *p*-value between quartile levels of each source of folate intake and racial groups in relation to PCa aggressiveness: 0.055 for DFE, 0.019 for Synthetic, 0.016 for Natural.

**Table 3:** Associations ^5^ between ingestion of dietary folate equivalents, synthetic and natural folate, and PCa aggressiveness among AA (*n* = 722) and EA (*n* = 775) ^4^.

	African Americans				European Americans			
	OR ^1^/95% CI ^2^	*p*-Value	Adjusted Mean	SE ^3^ of Adjusted Mean	OR/95% CI	*p*-Value	Adjusted Mean	SE of Adjusted Mean
**Dietary folate equivalents (DFE)**								
Q1 (<409.1 mcg/d)	1.00		0.33	0.048	1.00		0.33	0.045
Q2 (409.1–553.0 mcg/d)	1.22 (0.72, 2.09)	0.461	0.37	0.049	0.97 (0.59, 1.58)	0.895	0.32	0.044
Q3 (553.0–810.3 mcg/d)	1.03 (0.60, 1.79)	0.904	0.33	0.045	0.64 (0.38, 1.07)	0.090	0.24	0.039
Q4 (>810.3 mcg/d)	0.96 (0.51, 1.81)	0.903	0.32	0.049	0.45 (0.23, 0.89)	0.022	0.18	0.043
**Synthetic**								
Q1 (<92.6 mcg/d)	1.00		0.33	0.048	1.00		0.34	0.046
Q2 (92.6–150.2 mcg/d)	0.88 (0.52, 1.49)	0.630	0.30	0.043	0.77 (0.47, 1.25)	0.286	0.28	0.040
Q3 (150.2–243.1 mcg/d)	0.93 (0.54, 1.60)	0.795	0.32	0.043	0.54 (0.32, 0.90)	0.018	0.22	0.036
Q4 (>243.1 mcg/d)	1.39 (0.77, 2.51)	0.275	0.41	0.052	0.62 (0.32, 1.18)	0.145	0.24	0.052
**Natural**								
Q1 (<209.2 mcg/d)	1.00		0.29	0.047	1.00		0.34	0.045
Q2 (209.2–303.1 mcg/d)	1.50 (0.88, 2.58)	0.138	0.38	0.047	0.68 (0.42, 1.12)	0.129	0.26	0.040
Q3 (303.1–435.6 mcg/d)	1.49 (0.86, 2.58)	0.158	0.38	0.045	0.54 (0.32, 0.91)	0.022	0.22	0.037
Q4 (>435.6 mcg/d)	1.19 (0.62, 2.27)	0.602	0.33	0.051	0.79 (0.42, 1.49)	0.475	0.29	0.055

^1^ Odds ratio. ^2^ Confidence interval. ^3^ Standard error. ^4^ Interaction effect *p*-value between quartile levels of each source of folate intake and racial groups in relation to PCa aggressiveness: 0.245 for DFE, 0.144 for Synthetic, 0.033 for Natural. ^5^ Adjusted variables: age, BMI, previous screening, smoking status, education, first-degree relative diagnosed with PCa, total energy, and the residual (continuous) saturated fat intake.

## Data Availability

The data presented in this study are available on request from the corresponding author. The data are not publicly available due to confidential agreements in the original consent form.
